# Picrasidine I Triggers Heme Oxygenase-1-Induced Apoptosis in Nasopharyngeal Carcinoma Cells via ERK and Akt Signaling Pathways

**DOI:** 10.3390/ijms23116103

**Published:** 2022-05-29

**Authors:** Hsin-Yu Ho, Ping-Ju Chen, Yi-Ching Chuang, Yu-Sheng Lo, Chia-Chieh Lin, Ming-Ju Hsieh, Mu-Kuan Chen

**Affiliations:** 1 Oral Cancer Research Center, Changhua Christian Hospital, Changhua 500, Taiwan; 183581@cch.org.tw (H.-Y.H.); 177267@cch.org.tw (Y.-C.C.); 165304@cch.org.tw (Y.-S.L.); 181327@cch.org.tw (C.-C.L.); 2 Department of Dentistry, Changhua Christian Hospital, Changhua 500, Taiwan; 139370@cch.org.tw; 3 Institute of Medicine, Chung Shan Medical University, Taichung 402, Taiwan; 4 Department of Post-Baccalaureate Medicine, College of Medicine, National Chung Hsing University, Taichung 402, Taiwan; 5 Graduate Institute of Biomedical Sciences, China Medical University, Taichung 404, Taiwan; 6 Department of Otorhinolaryngology, Head and Neck Surgery, Changhua Christian Hospital, Changhua 500, Taiwan

**Keywords:** picrasidine I, apoptosis, mitogen-activated protein kinase (MAPK) pathway, heme oxygenase-1

## Abstract

Nasopharyngeal carcinoma (NPC) has a higher incidence in Taiwan than worldwide. Although it is a radiosensitive malignancy, cancer recurrence is still high in the advanced stages because of its ability to induce lymph node metastasis. Picrasidine I from *Picrasma quassioides* has been reported as a potential drug for targeting multiple signaling pathways. The present study aimed to explore the role of picrasidine I in the apoptosis of NPC cells. Our results show that picrasidine I induced cytotoxic effects in NPC cells and caused cell cycle arrest in the sub-G1, S, and G2/M phases. Western blot analysis further demonstrated that the modulation of apoptosis through the extrinsic and intrinsic pathways was involved in picrasidine I-induced cell death. Downregulation of the ERK1/2 and Akt signaling pathways was also found in picrasidine I-induced apoptosis. Additionally, the apoptosis array showed that picrasidine I significantly increased heme oxygenase-1 (HO-1) expression, which could act as a critical molecule in picrasidine I-induced apoptosis in NPC cells. The Cancer Genome Atlas (TCGA) and Gene Expression Omnibus (GEO) datasets also revealed that the *HMOX1* mRNA level (HO-1) is lower in patients with head and neck squamous carcinoma (HNSCC) and NPC than in patients without cancer. Our study indicated that picrasidine I exerts anticancer effects in NPC by modulating HO-1 via the ERK and Akt signaling pathways.

## 1. Introduction

Squamous carcinoma cells derived from mucosal epithelial cells in the larynx, pharynx, nasopharynx, and oral cavity are known to be the most common malignancies of the head and neck [1]. Among head and neck cancers, nasopharyngeal carcinoma (NPC) has a low incidence rate worldwide, but is endemic in North Africa, Southern Asia, and Southeast Asia. Because of the anatomic isolation of this tumor, NPC patients tend to be diagnosed at an advanced stage and exhibit a higher rate of cervical lymph node metastasis [2]. In clinical treatment, NPC is a radiosensitive malignancy in which patients have a survival rate of 60% in stage I and stage II [3]; however, the median survival time in the advanced stages is 3 years [4]. The high mortality in the advanced stage highlights the urgent need to evaluate anticancer agents for NPC.

Chemotherapeutic agents are used to inhibit the proliferation of uncontrolled cancer cells, reduce symptoms, or prolong life in cancer treatment. The way in which chemotherapeutic agents exert their anticancer effect is mostly by targeting apoptosis in cancer cells [5]. Apoptosis, a type of programmed cell death, is morphologically classified by chromatin condensation, DNA fragmentation, cell shrinkage, and membrane blebbing [6]. Caspases (cysteine-aspartic proteases), a family of cysteine proteases that provides critical links in cell death, activate each other through the cleavage of different substrates in the nucleus or cytoplasm, resulting in apoptotic cell death [7]. The activation of caspases can be launched through the cellular membrane (death receptor pathway) or through the mitochondria (mitochondrial pathway). Through the stimulation of the Fas receptor (CD95, a member of the tumor necrosis factor receptor superfamily 6) or tumor necrosis factor (TNF) apoptosis-inducing ligand (TRAIL), activated caspase-8 can then initiate downstream signals such as caspase-3 [8]. The Bcl-2 family, including pro-apoptosis proteins (such as Bax and Bak) and anti-apoptosis proteins (such as Bcl-2 and Bcl-xL), were involved in the mitochondrial pathway and regulated the release of caspase activators [9].

Natural products are considered to be important sources of new chemical substances and new drugs. Various kinds of natural compounds, such as polyphenols [10], flavonoids [11], and alkaloids [12], are known to exert anticancer properties. Alkaloids are secondary metabolites for plants’ defensive mechanisms that contain ring structures and nitrogen atoms [13]. Naturally derived alkaloids, such as taxol and camptothecin, are well known for their anticancer properties that target DNA replication and result in apoptosis [14]. Alkaloids such as picrasidine I, picrasidine G, picrasidine Q, and picrasidine N derived from *Picrasma quassioides* have been reported to have anti-inflammatory, anticancer, and peroxisome proliferator-activated receptor (PPAR) β/δ agonist properties [15,16,17,18]. Both picrasidine N and picrasidine C were found to be subtype-selective PPARβ/δ agonists and targeted different PPAR genes in Zhao’s research [18,19]. Picrasidine N decreased cell viability by modulating the EGFR/STAT3 signaling pathway in EGFR-overexpressing triple-negative breast cancer cells [16]. Furthermore, recent studies showed that picrasidine I suppressed osteoclastogenesis by attenuating ROS production and induced apoptosis in oral cancer cells [15,20]. However, the anticancer mechanism of picrasidine I in NPC has not been investigated. Here, we aimed to investigate the effects of picrasidine I on NPC and elucidated the mechanism underlying the induction of cell apoptosis in present study.

## 2. Results

### 2.1. Picrasidine I Decreased the Cell Viability of NPC Cell Lines and Caused Cell Accumulation in Sub-G1, S, and G2/M Phases 

**Figure 1:**
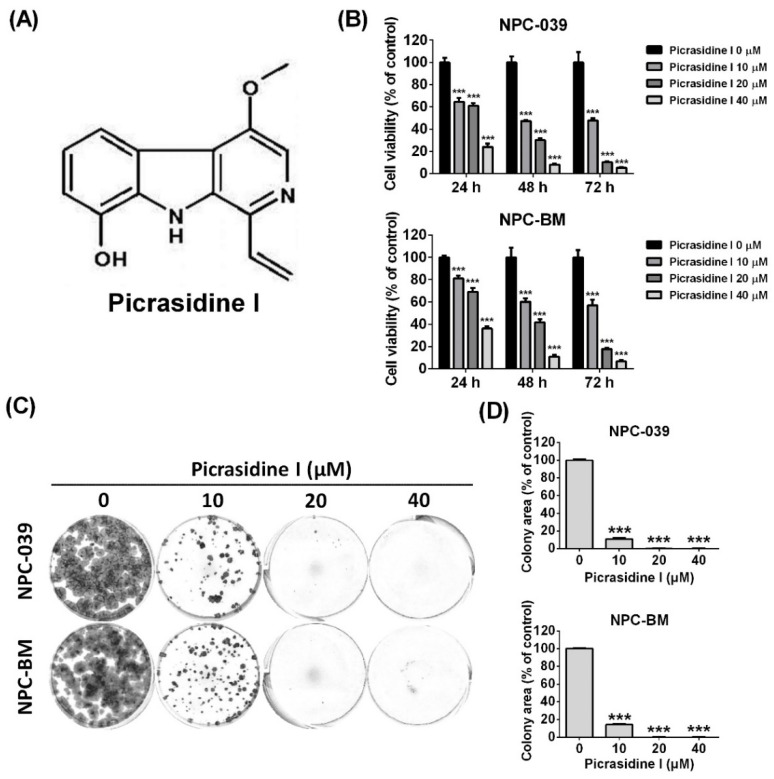
Picrasidine I-induced cell death in NPC cells. (**A**) Structure of picrasidine I. (**B**) Human NPC cell lines (NPC-039 and NPC-BM) were treated with different doses of picrasidine I (0, 10, 20, and 40 µM) for 24, 48, and 72 h, respectively. The cell viability was measured using an MTT assay. (**C**,**D**) NPC-039 and NPC-BM cell lines were cultured with different doses of picrasidine I (0, 10, 20, and 40 µM) for 14 days. Fresh culture medium was replaced every 3 days. *** *p* < 0.001 compared with the vehicle treatment group.

**Figure 2:**
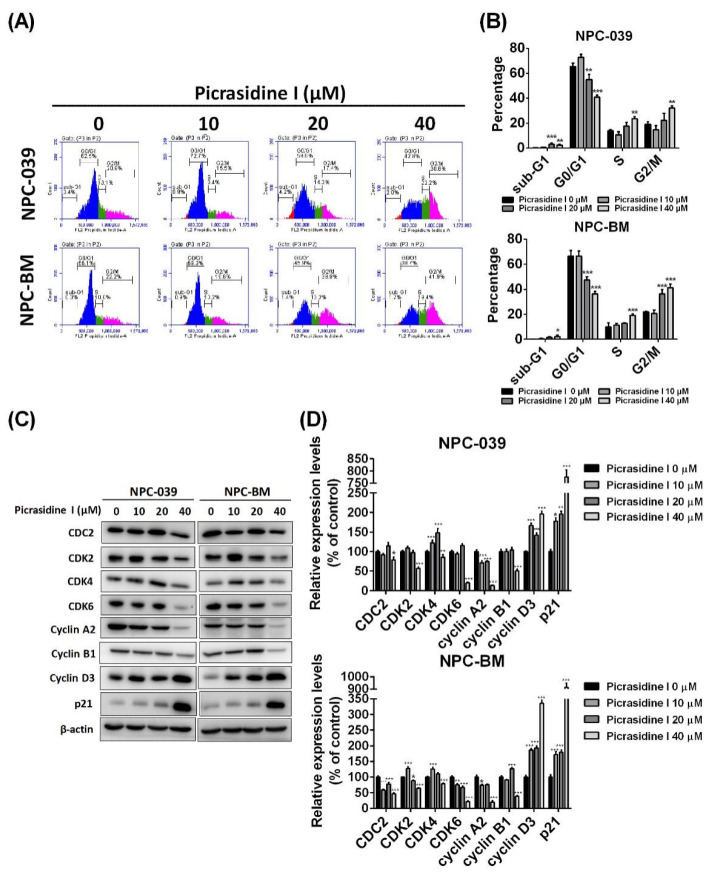
Picrasidine I caused NPC cell cycle arrest in the sub-G1, S, and G2/M phases. (**A**,**B**) The cells were stained with PI staining buffer and cell distribution was analyzed using flow cytometry and divided into four phases (sub-G1, G0/G1, S, and G2/M phases). (**C**,**D**) Western blot of proteins associated with the cell cycle was performed; β-actin was used as an internal control. * *p* < 0.05, ** *p* < 0.01, and *** *p* < 0.001 compared with the vehicle treatment group.

To detect the effect of picrasidine I (Figure 1A) on NPC cell lines, the dose–response curve of picrasidine I was first analyzed using an MTT assay. Figure S1 shows that the IC_50_ values of picrasidine I in NPC-039 and NPC-BM were 23.76 µM and 30.02 µM, respectively. We therefore chose doses of picrasidine I from 10 µM to 40 µM that resulted in low toxicity and high toxicity of picrasidine I in NPC cell lines. The indicated doses of picrasidine I (10, 20, and 40 µM) were then treated to NPC-039 and NPC-BM cells for 24, 48, and 72 h. The results show that picrasidine I decreased the viability of both NPC cell lines in a dose- and time-dependent manner (Figure 1B). Meanwhile, colony formation analysis (Figure 1C,D) demonstrated a similar result to the MTT assay, providing evidence on of short-term and long-term cytotoxicity of picrasidine I in NPC cell lines. The cell cycle system is an important mechanism that is responsible for cell duplication and is regulated by cyclin-dependent kinases (CDKs) and cyclins [21]. The distribution of NPC cells treated with picrasidine I was found to be decreased in the G0/G1 phase and accumulated in the S and G2/M phases (Figure 2A). Cells that were slightly accumulated in the sub-G1 phase were also found in both NPC cell lines (Figure 2B). Protein levels of CDC2 (also known as CDK1), CDK2, CDK4, CDK6, cyclin A2, and cyclin B1 were suppressed by 40 µM of picrasidine I treatment, while cyclin D3 and p21 were induced after picrasidine I treatment (Figure 2C,D). Interestingly, the protein levels of CDK2 and CDK4 were upregulated by.a low dose of picrasidine I (10 or 20 µM). The results showed that S and G2/M phase arrest might be due to a high dose of picrasidine I (40 µM). These results suggest that picrasidine I has the potential to exert toxicity toward NPC cells and cause cell accumulation in the sub-G1, S, and G2/M phases.

### 2.2. Picrasidine I Induced Apoptosis in NPC Cells via Extrinsic and Intrinsic Pathways

**Figure 3:**
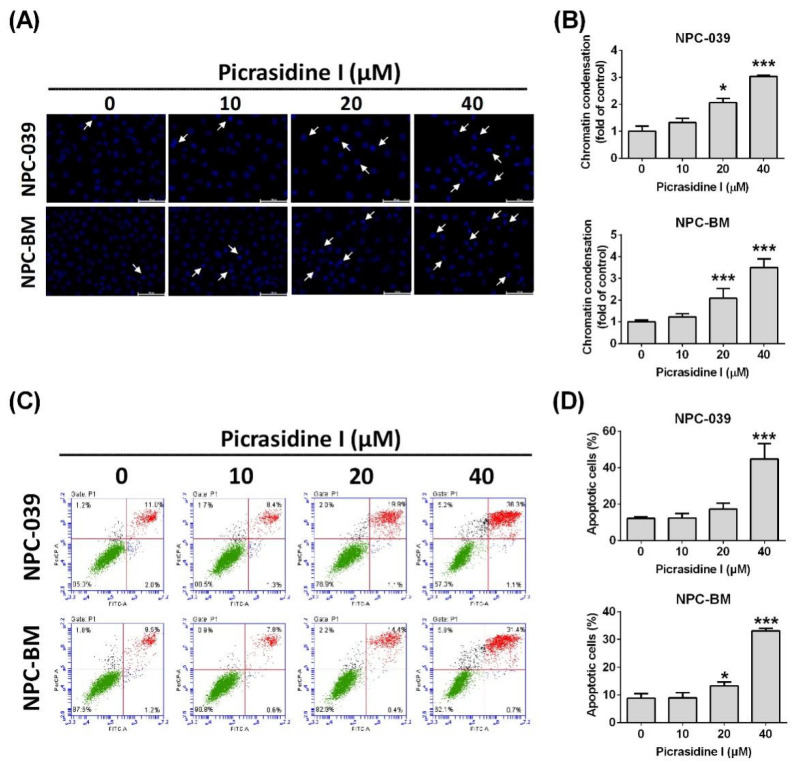
The effect of picrasidine I on apoptosis in NPC cells. (**A**,**B**) Cells were incubated with different doses of picrasidine I (0, 10, 20, and 40 µM) for 24 h. Apoptosis was determined with DAPI staining by fluorescence microscopy. The arrows indicate condensed cells. Scale bar = 100 µm. (**C**,**D**) Apoptotic cells were stained with annexin V and PI staining buffers and measured using flow cytometry. Annexin V (−)/PI (−) indicate live cells; annexin V (+)/PI (−) indicate early apoptotic cells; annexin V (+)/PI (+) indicate late apoptotic cells; annexin V (−)/PI (+) indicate early apoptotic cells. FITC-A: annexin V, PerCP-A: PI. The apoptotic cells included early and late apoptotic cells. * *p* < 0.05 and *** *p* < 0.001 compared with the vehicle treatment group.

**Figure 4:**
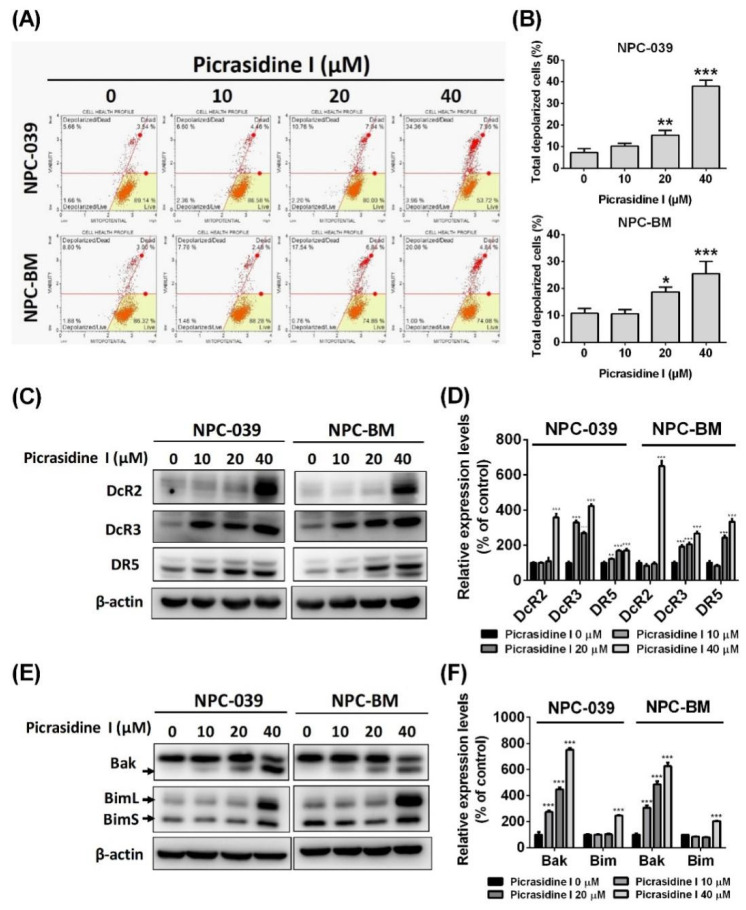
Picrasidine I induced apoptosis in NPC cells by means of extrinsic and intrinsic pathways. (**A**,**B**) Picrasidine I-treated cells (0, 10, 20, and 40 µM) were stained with Muse MitoPotential dye and analyzed by flow cytometry. Total depolarized cells contained live cells and dead cells. (**C**,**D**) The expression levels of death receptors (DcR2, DcR3, and DR5) were analyzed by the Western blot assay, with β-actin used as an internal control. (**E**,**F**) Western blot of intrinsic pathway-related proteins, namely Bak and Bim (including isoforms BimL and BimS), was performed. β-actin was used as an internal control. * *p* < 0.05, ** *p* < 0.01, and *** *p* < 0.001 compared with the vehicle treatment group.

To further verify whether picrasidine I decreased NPC cell viability by inducing cell apoptosis, we subsequently performed condensed chromatin analysis, annexin V/PI stain analysis and mitochondrial membrane potential analysis. Figure 3A,B shows that the condensation level of DAPI-stained chromatin, which is a morphological hallmark of apoptosis [22], increased after high doses of picrasidine I treatment (20 and 40 µM) in NPC-039 and NPC-BM cells. Picrasidine I-induced apoptotic cells stained by annexin V and PI are illustrated in Figure 3C and reached nearly 40% and 30% after treatment with the highest dose of picrasidine I in NPC-039 and NPC-BM cells, respectively (Figure 3D). The loss of the electrochemical gradient (Δψ) and the depolarization of mitochondria are universally associated with apoptosis [23]. Thus, we then measured the dynamics of mitochondrial membrane potential using Muse MitoPotential dye. As shown in Figure 4A, the distribution of cells was transferred from the polarized/live part (yellow area) to the depolarized/dead part as the dose of picrasidine I increased. The total proportion of depolarized cells was raised to almost 36% and 25% after treatment with 40 µM picrasidine I in NPC-039 and NPC-BM cells, respectively (Figure 4B). 

**Figure 5:**
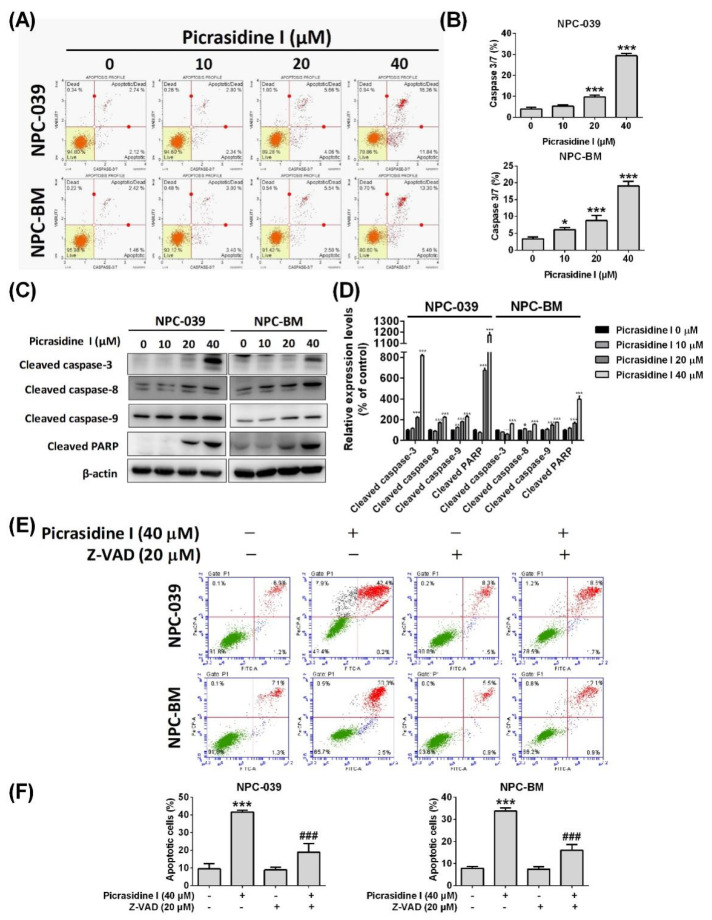
The caspase cascades induced by picrasidine I in NPC cells. (**A**,**B**) Cells were collected after being treated with different doses of picrasidine I and stained with Muse™ Caspase 7-AAD reagent and Muse™ Caspase-3/7 reagent. The percentages of caspase3/7-containing apoptotic cells and apoptotic/dead cells were measured using a Muse cell analyzer flow cytometer. (**C**,**D**) The expression levels of cleaved caspase-3, -8, -9, and PARP were analyzed by Western blot assays, with β-actin used as an internal control. (**E**,**F**) Cells treated with picrasidine I or Z-VAD were stained with annexin V and PI staining buffers and measured using flow cytometry. Recovered apoptotic cells were found in the co-treatment group. * *p* < 0.05, ** *p* < 0.01, and *** *p* < 0.001 compared with the vehicle treatment group. ^###^ *p* < 0.001 compared with the picrasidine I only treatment group.

The processes of apoptosis involve complex molecule mechanisms including an extrinsic pathway (known as the death receptor pathway) and an intrinsic pathway (known as the mitochondrial pathway) that activate cell signal cascades. Our results show that protein levels of DcR2, DcR3, and DR5 were significantly increased after picrasidine I treatment (Figure 4C,D). The pro-apoptotic proteins, such as Bak and Bim, were also found to be elevated in picrasidine I-treated NPC cells (Figure 4E,F). The activation of caspase (cysteine-aspartic protease) cascades relays the signals from extrinsic and intrinsic pathways. Using flow cytometry, we found that the activation of caspase 3/7 was elevated after picrasidine I treatment (Figure 5A,B). Moreover, the protein levels of cleaved caspase-3, -8, -9, and PARP, as measured by the Western blot assay, were increased after treatment with a high dose of picrasidine I (Figure 5C,D). 

To confirm whether picrasidine I-induced apoptosis progresses through extrinsic or intrinsic pathway-activated caspases, the pan-caspase inhibitor Z-VAD was administered to picrasidine I-treated NPC cells (Figure 5E,F). In the annexin V/PI assay, the apoptotic cells treated with Z-VAD and picrasidine I were observed to be significantly recovered compared with the picrasidine I treatment alone group. The results suggest that picrasidine I induced NPC cell apoptosis via activation of caspase cascades.

### 2.3. The Activation of Mitogen-Activated Protein Kinases (MAPKs) and Akt Signaling Pathways Is Involved in Picrasidine I-Induced Apoptosis

**Figure 6:**
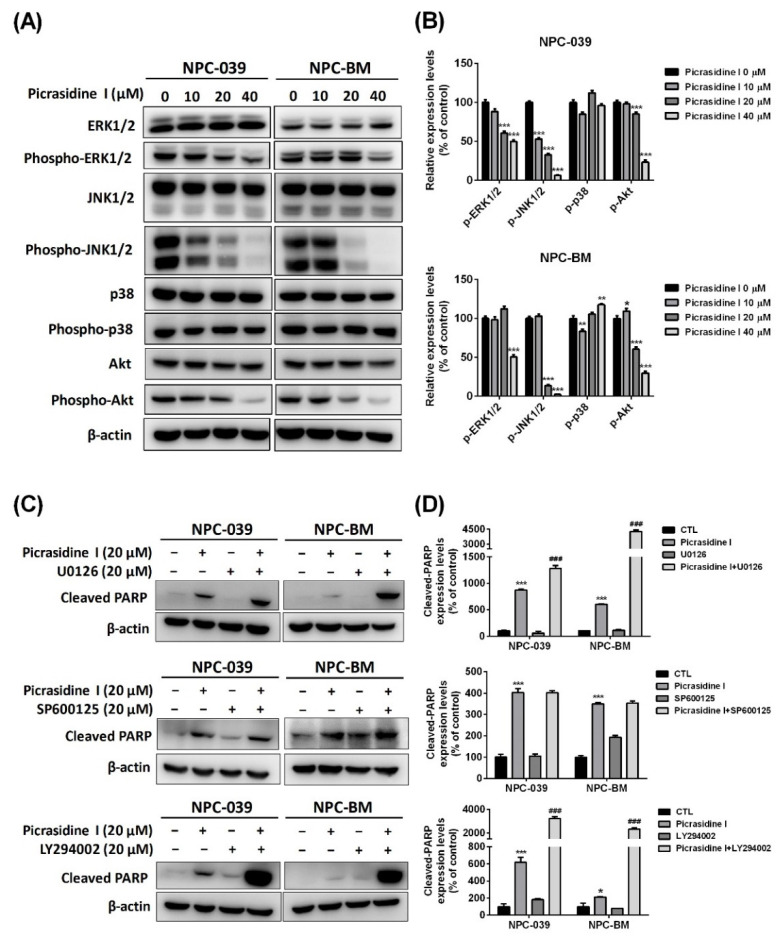
Picrasidine I regulated apoptosis via ERK and Akt signaling. (**A**,**B**) The expression levels of proteins of the MAPK and Akt pathways were analyzed by the Western blot assay, with β-actin used as an internal control. (**C**,**D**) Cells treated with picrasidine I or MAPK inhibitors were analyzed by Western blot assay, with β-actin used as an internal control. * *p* < 0.05, ** *p* < 0.01, and *** *p* < 0.001 compared with the vehicle treatment group. ^###^ *p* < 0.001 compared with the picrasidine I only treatment group.

MAPKs and Akt signaling are chains of proteins that communicate signals from the receptors of the cell membrane to the inner cell and function by modulating transcription factors that affect gene expression [24,25]. These signaling pathways are also involved in various biological responses, such cell proliferation, apoptosis, and autophagy [25,26,27]. Here, we performed Western blot analysis of JNK1/2, ERK1/2, p38 MAPK, and Akt, and evaluated the pathway through which picrasidine I regulates cell apoptosis. As shown in Figure 6A,B, phosphorylated ERK1/2, JNK1/2, and Akt were significantly decreased after treatment with different doses of picrasidine I in both NPC-039 and NPC-BM cells. To further confirm the signals that are involved in picrasidine I-induced apoptosis, ERK1/2 inhibitor (U0126), JNK1/2 inhibitor (SP600125), and PI3k/Akt inhibitor (LY294002) were applied. The result demonstrate that the expression of cleaved PARP is significantly higher in the picrasidine I combined with U0126 group and the picrasidine I combined with LY294002 group than in the picrasidine I only treatment group (Figure 6C,D). The addition of MAPK inhibitors and the PI3k/Akt inhibitor indicated that picrasidine I might regulate NPC cell apoptosis by activating the ERK1/2 and Akt signaling pathways. 

### 2.4. HO-1 Participates in Picrasidine I-Induced Apoptosis via Activating the ERK1/2 and Akt Signaling Pathways

**Figure 7:**
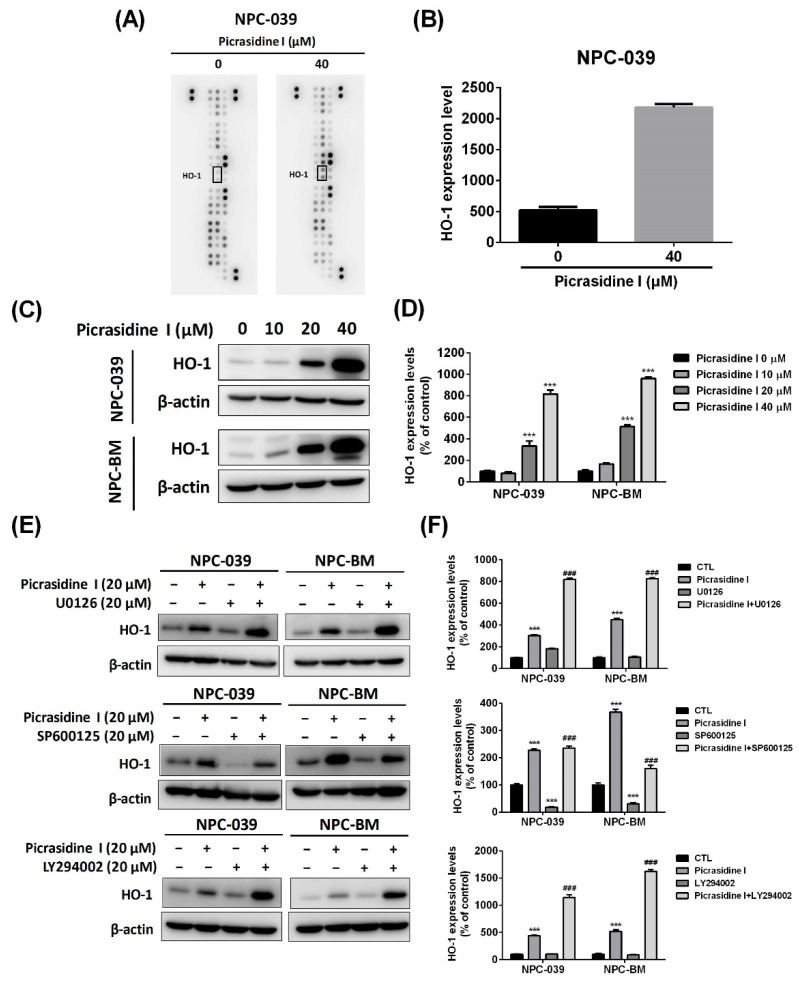
Picrasidine I induced HO-1 expression via ERK and Akt signaling. (**A**,**B**) The image of the Human Apoptosis Array with 35 apoptosis associated proteins was applied to picrasidine I-treated NPC-039 cells. The protein level of HO-1 was quantitated using ImageJ. (**C**,**D**) Cells were incubated with different doses of picrasidine I (0, 10, 20, and 40 µM) for 24 h. The expression level of HO-1 was analyzed by Western blot assay, with β-actin used as an internal control. (**E**,**F**) Cells treated with picrasidine I or MAPK inhibitors were analyzed by Western blot assay. The expression level of HO-1 was measured, with β-actin used as an internal control. *** *p* < 0.001 compared with the vehicle treatment group. ^###^ *p* < 0.001 compared with the picrasidine I only treatment group.

To elucidate possible molecules that participated in picrasidine I-induced apoptosis, a Human Apoptosis Array kit was used for analysis. The top five differentially apoptotic-related proteins, including up- and down-regulated proteins, are listed in Figure S2. For the Human Apoptosis Array kit, we found that picrasidine I at a dose of 40 µM increased HO-1 expression most in NPC-039 cells compared with other proteins (Figure 7A,B). HO-1 is an essential enzyme for heme metabolism, and cleaves heme into carbon monoxide, biliverdin, and ferrous iron [28]. Evidence is increasing that HO-1 plays a critical role in several cancers, such as oral cancer [29,30], lung carcinoma [31], and breast cancer [32]. Therefore, we then investigated the role of HO-1 in our study. Western blot analysis proved that picrasidine I induced the protein expression of HO-1 in a dose-dependent manner (Figure 7C,D). Through the combination of a signaling pathway inhibitor and picrasidine I at a dose of 20 µM, the expression level of HO-1 was significantly elevated in the picrasidine I combined with U0126 group and the picrasidine I combined with LY294002 group compared to the picrasidine I only treatment group (Figure 7E,F). The results indicate that picrasidine I induced HO-1 expression through the ERK1/2 and Akt signaling pathways, but not the JNK1/2 signaling pathway. 

**Figure 8:**
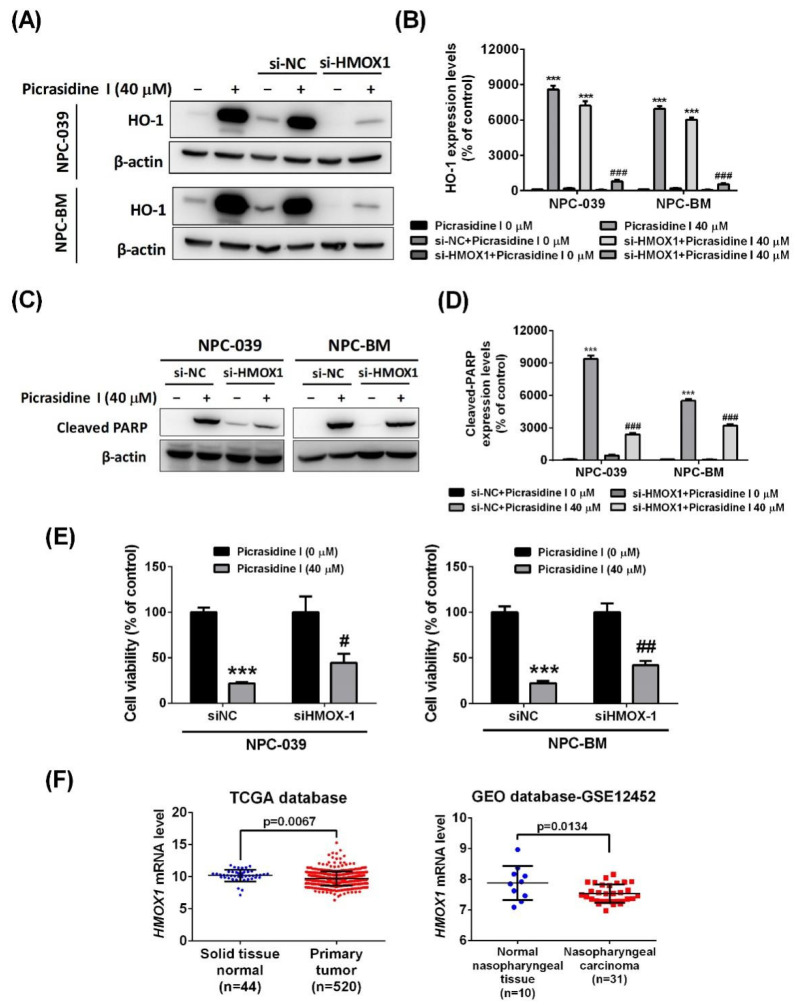
HO-1 is involved in picrasidine I-induced apoptosis. (**A**,**B**) The effect of the combination of the effect *HMOX1* siRNA and picrasidine I on protein level of HO-1 was analyzed by Western blot assay, with β-actin used as an internal control. (**C**,**D**) The effect of the combination of *HMOX1* siRNA and picrasidine I on protein level of cleaved PARP was analyzed by Western blot assay, with β-actin used as an internal control. (**E**,**F**) The cell viability of NPC-039 and NPC-BM was measured by MTT assay after treatment with either *HMOX1* siRNA and picrasidine I. (**F**) The *HMOX1* mRNA level of solid tissue normal and primary tumor were retrieved from HNSCC dataset of TCGA database (left panel). The *HMOX1* mRNA expression level in normal nasopharyngeal tissue and NPC were retrieved from GSE12452 of the GEO database (right panel). *** *p* < 0.001 compared with the vehicle treatment group. ^#^ *p* < 0.05, ^##^ *p* < 0.01, and ^###^ *p* < 0.001 compared with the picrasidine I only treatment group.

Furthermore, to verify the role of HO-1 in picrasidine I-induced apoptosis, we involved *HMOX1* (the gene name of HO-1) siRNA in the analysis. The results show that the *HMOX1* siRNA significantly inhibited picrasidine I-induced HO-1 protein expression (Figure 8A,B) and picrasidine I-induced cleaved PARP (Figure 8C,D). Moreover, the picrasidine I inhibited the cell viability of both NPC-039 and NPC-BM cells, which was slightly recovered by combining picrasidine I and *HMOX1* siRNA (Figure 8E,F). These findings indicate that HO-1 is involved in picrasidine I-regulated apoptosis in NPC cells.

Bioinformatics also provided evidence on the role of HO-1 in head and neck cancer. After analysis of 44 normal tissues and 520 primary tumor tissues of HNSCC from the TCGA database, and we found a lower expression level of *HMOX1* mRNA in tumor tissues than normal tissues (Figure 8F). In addition, a similar result was found in GSE12452 from the GEO database, in that the mRNA level of *HMOX1* was lower in nasopharyngeal carcinoma tissues than normal nasopharyngeal tissues. Overall, our findings suggest that the increasing level of HO-1 is critical for picrasidine I-induced cell death, and that it might be a biomarker of NPC.

## 3. Discussion

Picrasidine I is a β-carboline alkaloid and has been more extensively explored in disease therapy than other picrasidines [15,20]. However, the mechanism of picrasidine I in cancer treatment still needs to be extensively explored. Our study provides evidence on the anticancer effect of picrasidine I, showing that the typical morphological features such as chromatin condensation and the loss of mitochondria membrane potential are found in picrasidine I-treated NPC cells. Picrasidine I not only induced cell cycle arrest in the sub-G1, S, and G2/M phases and regulated apoptosis-related proteins, but also decreased ERK and Akt signaling pathways in NPC cells. Moreover, the expression of HO-1 was proved to have a crucial role in picrasidine I-induced apoptosis in this study. 

The cell cycle is a process that involves the transition through cell phases, including G1, S, and G2, and mitosis, and is regulated by the activation and inactivation of CDK family proteins. The activation and inactivation of CDK-cyclin complexes were controlled by p53 regulated p21, which performed the role of a tumor suppressor and induced growth arrest [33]. The cell arrest effects caused by anticancer drugs are varied due to the different effects of anticancer drugs and cancer characteristics. Previous studies have showed that dehydrocrenatidine induced cell cycle arrest in the G2/M phase, and inhibited the protein level of cyclin A, cyclin B, CDK2, CDK4, and CDK6 in oral cancer cells [34]; cell cycle arrest in the S and G2/M phases was also found in picrasidine I-treated oral cancer cells and led to apoptosis [20]; and picrasidine G increased the percentage of the sub-G1 phase in breast cancer [16]. The naturally occurring bioflavonoid galangin has been documented to induce S phase arrest through inhibiting the PI3K/Akt signaling pathway in NPC cells [35]. A recent study also indicated that curcumin exerted anticancer effects on G2/M phase arrest and p21 elevation in NPC cells [36]. This evidence is in line with our study, which demonstrated that picrasidine I caused sub-G1, S, and G2/M phase arrest and also regulated the protein level of CDKs, cyclins, and p21 in NPC-039 and NPC-BM cells.

The activation of caspase cascades is involved in apoptosis, including extrinsic and intrinsic pathways. The extrinsic pathway is initiated by the combination of death receptors and their natural ligands, called the TNF family, while the intrinsic pathway is regulated by anti-apoptotic and pro-apoptotic proteins in the mitochondria [37,38]. In oral cancer, Liu et al. mentioned that picrasidine I induced apoptosis through upregulating death receptor proteins and the pro-apoptotic proteins Bak and Bim [20]. Picrasidine Q, with a similar molecule structure to picrasidine I, also showed an apoptotic effect by activating cleaved caspase-3, -7, and PARP in esophageal squamous cell carcinoma [17]. A natural product, brevilin A, with an apoptosis-inducing effect, was found to induce the protein expressions of Bax, cleaved caspase-9, and PARP, and reduce Bcl-2 protein level in NPC cells [39]. In order to explore the apoptotic mechanism of picrasidine I in NPC cells, NPC-039 and NPC-BM cells were treated with different doses of picrasidine I and analyzed using Western blot. The above studies supported our results that picrasidine I induced apoptosis through both the extrinsic and intrinsic pathways by upregulating the expression of DcR2, DcR3, DR5, Bak, Bim, and caspase cascades in NPC cells.

The MAPKs and Akt signaling pathways are important to link extracellular signals to the inner machinery that controls fundamental cellular processes including proliferation, growth, apoptosis, differentiation, and migration. Abnormal MAPK and Akt signals affect most of these processes and play a crucial role in cancer development [40]. For NPC cells, our previous studies have shown that the anticancer drug luteolin-7-O-glucoside modulated proliferation and apoptosis through the Akt signaling pathway [41], and that celastrol caused cell cycle G2/M phase arrest and apoptosis via ERK and p38 signaling pathways [42]. Treatment with picrasidine I in bone marrow macrophages demonstrated that picrasidine I suppressed RANKL-induced phosphorylated ERK, p38, and JNK [15]. Moreover, a recent study indicated that picrasidine I regulated cleaved PARP via the JNK signaling pathway in oral cancer [20]. The Western blot data of our study reported that picrasidine I inhibited the expression of p-ERK1/2, p-JNK1/2, and p-Akt. The combination of picrasidine I and each inhibitor further clarified that picrasidine I upregulated the cleaved PARP level by ERK and Akt signaling pathways and caused the apoptosis in both NPC-039 and NPC-BM cell.

HO-1 belongs to the heat shock protein (HSP) family and is known as HSP32. It is a stress-induced isoform of heme oxygenase that is presents throughout the body and in responses to stimuli such as cellular injury, oxidative stress, and diseases [43]. HO-1 has been reported to act in a protective or detrimental role in different diseases, for instance in kidney injury, neurodegeneration, and cancer progression [44,45]. It also plays a cytoprotective role in various types of tumor cells. A previous study found that enhanced Nrf2/HO-1 signaling may contribute to breast cancer progression, and miR-140-5p could be a strategy for nuclear factor erythroid 2-related factor 2 (Nrf2)-driven cancer progression [46]. In KRAS mutant colorectal cancer, Yang et al. revealed that cetuximab, an approved chemotherapeutic agent for metastatic colorectal cancer, promoted RSL3-induced ferroptosis by suppressing the p38/Nrf2/HO-1 signaling pathway [47]. In contrast, the expression of HO-1 in human malignancies indicated its effects on anti-tumor growth, anti-metastasis, and anti-angiogenesis, and even contributed to the chemo- and radical-resistance of cancer cells [48,49,50]. In OSCC, a high expression of HO-1 was detected in patients with well-differentiated SCC, and was negatively correlated with lymph node metastasis [51]. Our previous study also mentioned that chrysosplenol D elevated HO-1 level in oral cancer cells, and thereby induced cell apoptosis [30]. In the present study, we found that picrasisdine I induced the HO-1 level, and directly triggered apoptosis in both NPC-039 and NPC-BM cells. Moreover, the combination of picrasidine I and MAPK inhibitors revealed that picrasidine I induced HO-1 by means of the ERK and Akt signaling pathways. The TCGA database and GEO databases also indicated lower expression of *HMOX1* in the patient group than the normal group, which pointed to the important role of HO-1 in NPC. Although we discovered that picrasidine I regulated the ERK/HO-1 and Akt/HO-1 axis in NPC, the comprehensive mechanism of picrasidine I still needs be explored. Additionally, further investigations of picrasidine I in animal tumor models are desirable.

Collectively, we first revealed that picrasidine I, derived from *Picrasma quassioides*, exerts the effects of decreasing the cell viability of NPC cells and causing cell cycle arrest in the sub-G1, S, and G2/M phases. Moreover, the activation of apoptosis via ERK/HO-1 and Akt/HO-1 by picrasidine I indicated that HO-1 might be a potential target for antitumor treatment. We believe that the exploration of picrasidine I can promote a new therapeutic strategy for NPC. 

## 4. Materials and Methods

### 4.1. Cell Lines and Culture Conditions 

The human NPC-039 and NPC-BM nasopharyngeal carcinoma cell lines were gifts from Dr. Jen-Tsun Lin, Hematology and Oncology, Changhua Christian Hospital. NPC-039 was derived from the nasopharyngeal tumor of a 64-year-old Chinese male [52], whereas NPC-BM cells were derived from a bone marrow biopsy of a female Taiwanese patient with NPC [53]. Both NPC cell lines were cultured in RPMI-1640 medium (Gibco BRL; Grand Island, NY, USA) supplemented with 10% fetal bovine serum and 1% penicillin/streptomycin at 37 °C in a humidified incubator with 5% CO_2_.

### 4.2. Cell Viability Assay

The cell viability assay was conducted using 3-(4, 5-dimethylthiazol-2-yl) -2, 5-diphenyltetrazolium bromide (MTT; Sigma Aldrich, St. Louis, MO, USA). NPC-039 and NPC-BM cells were seeded into a 96-well plate at a density of 1 × 10^4^ cells per well and exposed to the indicated dose of picrasidine I (0, 10, 20, 40 µM) for 24, 48, or 72 h, respectively. The cells in the vehicle group (0 µM) were treated with 0.1% dimethyl sulfoxide (DMSO; Sigma Aldrich). Subsequently, MTT was added to each well and incubated with cells for 3 h at 37 °C. Formazan accumulated in the wells was dissolved with DMSO and measured at a wavelength of 570 nm using a microplate reader (BioTek, Winooski, VT, USA). In a further experiment, cells were transfected with human small-interfering ribonucleic acids (siRNAs) for HO-1 and scrambled siRNA, prior to picrasidine I (40 µM) treatment.

### 4.3. Colony Formation Assay

The colony formation assay is a cell viability assay based on the growth of a single cell into a colony defined as consisting of at least 50 cells [54]. Briefly, NPC-039 and NPC-BM cells were seeded in a 6-well plate at a density of 500 cells per well, and treated with the indicated dose of picrasidine I (0, 10, 20, 40 µM). The culture medium was replaced every 3 days to maintain adequate nutrition. Colonies were fixed with 4% paraformaldehyde after 14 days of treatment. The colonies were then stained with 10% Giemsa staining solution (Sigma-Aldrich) and counted under a stereomicroscope.

### 4.4. Cell Cycle Analysis

NPC-039 and NPC-BM cells were seeded into a 6-well plate at a density of 3 × 10^5^ cells per well and exposed to the indicated dose of picrasidine I (0, 10, 20, 40 µM). After 24 h, cells were collected and fixed with 70% ice cold ethanol at −20 °C overnight. Subsequently, after ethanol was discarded from cells, the cells were incubated with propidium iodide (PI) staining solution (BD Biosciences, Franklin Lakes, NJ, USA) for 30 min in the dark at room temperature. The stained cells were analyzed using BD CSampler Plus software (version 1.0; BD Biosciences).

### 4.5. Western Blot Analysis

Whole cell lysates that treated with indicated doses of picrasidine I were analyzed using a Western blot assay [55]. All treatment time of picrasidine I through this study was 24 h. Briefly, the appropriate amount of protein was separated by gel electrophoresis, then transferred to polyvinylidene fluoride membranes and blocked with 5% skim milk dissolved in Tris-buffered saline/Tween-20 buffer. The membranes were then incubated with the indicated primary antibody (antibodies against CDC2, CDK2, CDK4, CDK6, cyclin A2, cyclin B1, cyclin D3, p21, DcR2, DcR3, DR5, Bak, Bim, cleaved PARP, cleaved caspase-3, -8, -9, ERK1/2, phospho-ERK1/2, JNK1/2, phospho-JNK1/2, p38 MAPK, phospho-p38 MAPK, AKT, phospho-AKT, and HO-1 were purchased from Cell Signaling, Danvers, MA, USA; the antibody against β-actin was purchased from Novus Biologicals, Centennial, CO, USA) at 4 °C overnight. The membranes were washed and incubated with the relevant horseradish peroxidase (HRP)-conjugated secondary antibody (Jackson ImmunoResearch Laboratories Inc., West Grove, PA, USA). The membranes were visualized using a Western blot detection kit (Advansta Inc., San Jose, CA, USA) and measured by ImageQuant LAS 4000 Mini (GE Healthcare Life Sciences, Boston, MA, USA).

### 4.6. Nuclear Morphology Analysis

Cells stained with 4,6-diamidino-2-phenylindole (DAPI) dye (Sigma Aldrich) can be used to visualize nuclear changes and evaluate apoptosis [56]. NPC-039 and NPC-BM cells were treated with the indicated doses of picrasidine I (0, 10, 20, and 40 µM) for 24 h. Then, the cells were fixed with 4% paraformaldehyde and stained with DAPI dye (50 mg/mL) for 10 min. The blue fluorescence of stained cells was visualized using fluorescence microscopy (Leica Biosystems Division of Leica Microsystems Inc., Buffalo Grove, IL, USA).

### 4.7. Apoptosis Analysis

NPC-039 and NPC-BM cells were seeded into a 6-well plate at a density of 3 × 10^5^ cells per well and exposed to the indicated dose of picrasidine I (0, 10, 20, 40 µM) for 24 h. After 24 h, cells were collected and incubated with annexin V–fluorescein isothiocyanate solution and PI solution (BD Biosciences) for 20 min in the dark at room temperature. The percentage of apoptotic cells was analyzed using the BD CSampler Plus software (version 1.0; BD Biosciences).

### 4.8. Mitochondrial Membrane Potential Analysis

Mitochondrial membrane potential analysis has previously been described [57]. Briefly, cells that were treated with the indicated dose of picrasidine I were collected and stained with Muse MitoPotential dye (Luminex, Austin, TX, USA). The stained cells were measured using the Muse cell analyzer flow cytometer (MilliporeSigma, Burlington, MA, USA) and analyzed using Muse Cell Soft V1.4.0.0 Analyzer Assays (MilliporeSigma).

### 4.9. Caspase-3/7 Activity Assay

NPC-039 and NPC-BM cells were seeded into a 6-well plate at a density of 3 × 10^5^ cells per well and exposed to the indicated dose of picrasidine I (0, 10, 20, 40 µM). Subsequently, cells were collected and stained with Muse™ Caspase 7-AAD reagent and Muse™ Caspase-3/7 reagent (Luminex). The protocol was conducted according to the Muse™ Caspase-3/7 Kit user’s guide. The percentage of active caspase-3/7 cells was measured using the Muse cell analyzer flow cytometer (MilliporeSigma) and analyzed using Muse Cell Soft V1.4.0.0 Analyzer Assays (MilliporeSigma).

### 4.10. Proteome Profiler Analysis

The Proteome profiler analysis was conducted according to the manual for the Proteome Profiler Human Apoptosis Array kit (R&D Systems Inc., cat. ARY009; Minneapolis, MN, USA). Briefly, whole cell lysates were collected and incubated with membranes coated with antibodies at 4 °C overnight. Then, the membranes were washed and incubated with Streptavidin–HRP solution. The membranes were visualized using Chemi Reagent Mix and intensity was measured by ImageQuant LAS 4000 Mini (GE Healthcare Life Sciences).

### 4.11. RNA Interference Experiments

Human siRNAs for scrambled control and HO-1 were purchased from Cohesion Biosciences (London, UK). NPC-039 and NPC-BM cells were seeded into 6 cm dishes at a density of 6 × 10^5^ cells per well and transfected with each siRNA with Turbofect reagent (Thermo Fisher Scientific; Waltham, MA, USA). The effects of siRNA were further detected by Western blot analysis.

### 4.12. Bioinformatic Analysis

The Cancer Genome Atlas (TCGA) database and Gene Expression Omnibus (GEO) dataset GSE12452 were analyzed using GraphPad Prism V6.0 (GraphPad Software, Inc., CA, USA). From the TCGA, the head and neck squamous cell carcinoma (HNSCC) database, including 520 tumors cases and 44 normal cases, was selected to analyze the expression level of *HMOX1*. From the GEO dataset GSE12452, 10 cases of normal nasopharyngeal tissue and 31 cases of nasopharyngeal carcinoma were selected to analyze the expression level of *HMOX1*.

### 4.13. Statistical Analysis

All statistical measurements were performed using GraphPad Prism V6.0. One-way ANOVA with Tukey’s multiple comparisons test was used to examine the variation between control group and treatment groups in all experiments. Student’s *t*-test was used to examine the variation between normal and tumor tissues in the TCGA and GEO databases. A *p* value of <0.05, <0.01, and <0.001 was considered significant.

## Data Availability

All data generated or analyzed during this study are included in the present article.

## Supplementary Materials

The following are available online at https://www.mdpi.com/article/10.3390/ijms23116103/s1.
