# Supplementary material for: Picrasidine I Triggers Heme Oxygenase-1-Induced Apoptosis in Nasopharyngeal Carcinoma Cells via ERK and Akt Signaling Pathways
Source: Int J Mol Sci. 2022 May 29;23(11):6103. doi: 10.3390/ijms23116103 (PMC9181417; doi:10.3390/ijms23116103)
Supplement: Supplementary file 1 [file ijms-23-06103-s001.zip › ijms-1719666-supplementary.pdf]

Figure S1

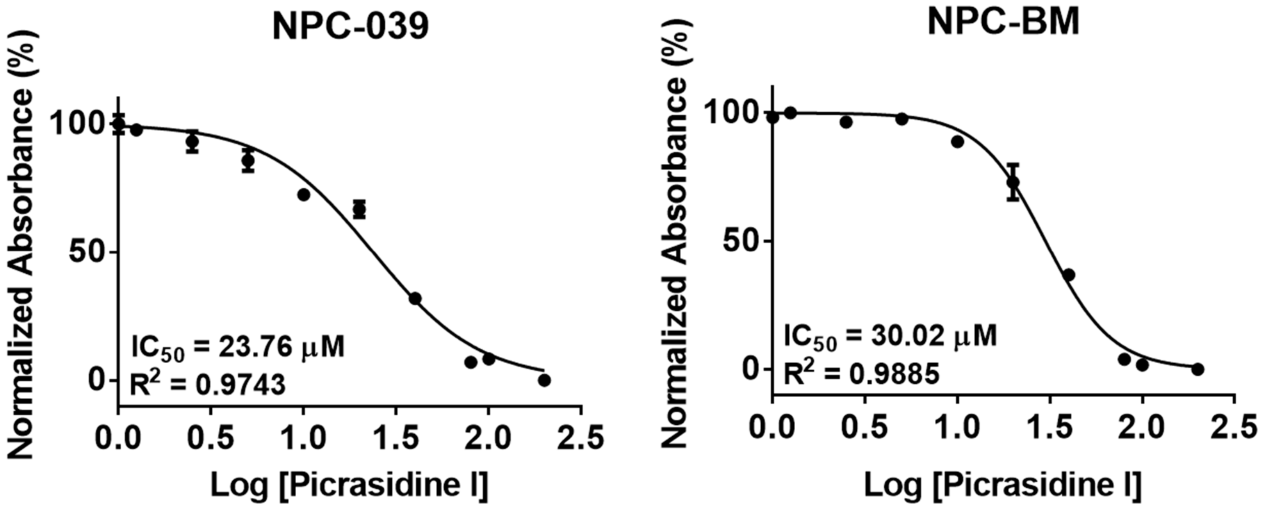

Figure S1. IC_{50} curve of picrasidine I in both NPC-039 and NPC-BM cell lines. Cells were treated with indicated doses of picrasidine I (0, 1.25, 2.5, 5, 10, 20, 40, 80, 100, and 200 μM) for 24 h. The IC_{50} values of picrasidine I were 23.76 μM and 30.02 μM in NPC-039 and NPC-BM cells, respectively. The data shown are means ± SE obtained from three independent experiments.

Figure S2

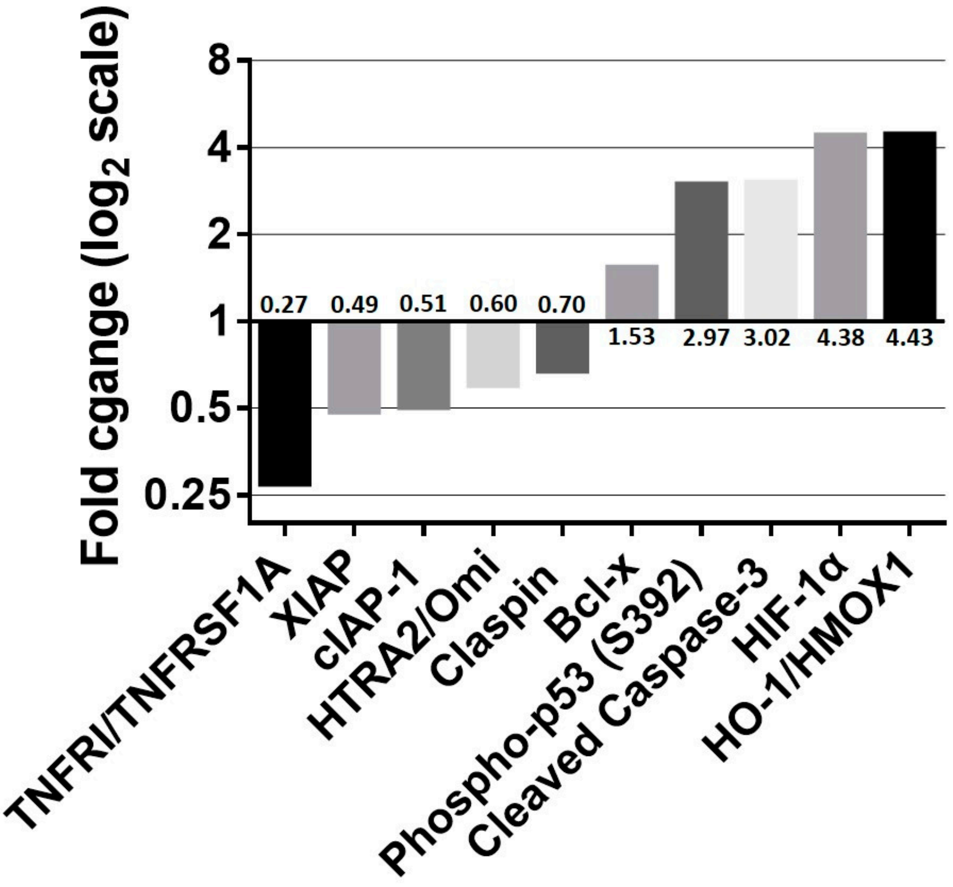

Figure S2. The fold change of apoptotic related proteins of NPC-039 cells. Cells treated with picrasidine I (40 μM) for 24 h were collected and analyzed by the Human Apoptosis Array kit. The top 5 up-regulated proteins (HO-1/HMOX1,

HIF-1α, Cleaved Caspase-3, Phosphor-p53 S392, and Bcl-x) and down-regulated proteins (TNFRI/TNFRSF1A, XIAP, cIAP, HTRA2/Omi, and claspin) were performed.
